# Breast Cancer Screening Using Clinical Breast Examination: A Cost-Effectiveness Analysis for South Africa

**DOI:** 10.1016/j.vhri.2025.101127

**Published:** 2025-09

**Authors:** Sithabiso D. Masuku, Olena Mandrik, Noreen D. Mdege, Gauravi Mishra, Richard Muwonge, Gesine Meyer-Rath, Naomi Lince-Deroche, Alan Brennan

**Affiliations:** 1 Health Economics and Epidemiology Research Office, Faculty of Health Sciences, University of the Witwatersrand, Johannesburg, South Africa; 2 SCHARR, Sheffield Centre for Health and Related Research, Division of Population Health, School of Medicine and Population Health, University of Sheffield, Sheffield, England, UK; 3 Department of Health Sciences, University of York, York, England, UK; 4 Centre for Research in Health and Development, York, England, UK; 5 Department of Preventive Oncology, Centre for Cancer Epidemiology, Homi Bhabha National Institute, Tata Memorial Centre, Mumbai, India; 6 Early Detection, Prevention and Infections Branch, International Agency for Research on Cancer, Lyon, France; 7 Department of Global Health, Boston University, Boston, MA, USA; 8 Independent Consultant, Athens, Greece

**Keywords:** breast cancer, clinical breast examination, cost-effectiveness analysis, screening

## Abstract

**Objectives:**

The World Health Organization emphasizes screening and early diagnosis to reduce advanced cancer incidence and mortality. In low-to-middle-income countries, breast cancer (BC) survival rates are low because of late detection. South Africa’s policy recommends twice-yearly clinical breast examinations (CBEs) for asymptomatic women aged 40 to 69. We assessed the impact of scaling up CBE screening on mortality and cost-effectiveness.

**Methods:**

Using trial data on downstaging, we compared the current baseline (5% coverage) with scenario 1 (25% coverage by year 5 [ie, 5% increase annually]) and scenario 2 (75% coverage by year 5, [ie, 17.5% increase annually]). A cohort model tracked women from screening to diagnosis, estimating downstaging’s impact on BC cases over their lifetime. Costs from the healthcare payer’s perspective are presented in 2022 US dollars.

**Results:**

Five-year screen detection rates were 2.39 and 2.08 per 1000 women screened for scenarios 1 and 2, respectively. Scenario 1 reduced BC mortality by 0.7% and scenario 2 by 2.3%. Compared with no screening, the current baseline screening program averts 1645 disability-adjusted life years (DALYs) at $20 341/DALY averted. Scenario 1 averted 3823 DALYs with economic efficiency improving to $17 776/DALY averted, whereas scenario 2 averted 12 165 DALYs at $19 552/DALY averted.

**Conclusions:**

CBE scale-up effectively saves life years but is not cost-effective under the country’s opportunity cost-derived threshold of $3015/DALY averted. However, decisions on the best screening policy are not solely based on cost-effectiveness. They involve careful consideration of budgetary constraints and competing healthcare priorities. Scale-up should consider system capacity, minimum care standards and cost-effective early detection strategies.

## Highlights

• The World Health Organization emphasizes screening and early diagnosis to reduce advanced cancer incidence and mortality. South Africa’s policy recommends biannual clinical breast examinations (CBE) for women aged 40 to 69, but the cost-effectiveness of CBE scale-up remains unclear.
• CBE scale-up effectively saves life years but is not cost-effective under the country’s opportunity cost-derived threshold of $3015/disability-adjusted life year averted.
• Decisions on best screening policy are not solely based on cost-effectiveness but involve consideration of budgetary constraints and competing healthcare priorities.

## Introduction

According to the World Health Organization (WHO), screening and early diagnosis are key to initiatives aimed at reducing the incidence of advanced cancer and mortality.^1^^,^^2^ Based on data from the Surveillance, Epidemiology, and the End Results Program in the United States, the 5-year relative survival rate for diagnosed breast cancer (BC) is 99.1% when a tumor has not yet metastasized but drops to 30% for metastasized disease.^3^ In low-to-middle-income countries (LMICs), the reported 5-year BC survival rate for localized cancer is >75% but that for metastatic cancer is as low as 15%.^4^^,^^5^ This strongly supports implementing early detection programs for BC in LMIC settings such as South Africa.

The implementation of an effective breast screening tool to identify asymptomatic individuals with abnormalities requiring further diagnostic investigations^6^ should decrease BC mortality by shifting diagnosis and treatment to earlier stages (downstaging); however, such a tool must be affordable. Studies in several high-income countries have shown that mammography, the current gold standard for screening in high-income countries, is the only BC screening modality that can reduce mortality.^7^, ^8^, ^9^, ^10^, ^11^ Unfortunately, mammography costs are prohibitive for most LMICs.^12^ In LMICs, where weak health systems with limited resources are common, and mammography is unavailable, a clinical breast examination (CBE), comprising a careful physical examination of the breasts of an asymptomatic woman by a doctor or other healthcare provider,^13^ is considered a promising approach by the WHO for the early detection of BC because of its low cost.^14^ In clinical trials conducted in Mumbai and Trivandrum, India, CBEs were shown to reduce the disease stage at diagnosis.^15^^,^^16^ Downstaging or shifting the majority of tumors from late stage to early stage increases survival rates because more treatment options are available and complexity is reduced.^17^ Together, these factors contribute to improved patient outcomes and lower healthcare costs.

In South Africa, stage 3 BC, which is considered late-stage cancer, is the most common stage at presentation.^18^^,^^19^ Since 2017, the South Africa BC Control Policy (BCCP) has recommended twice-yearly CBEs for asymptomatic women aged 40 to 69 years each time they present to a public-sector primary healthcare clinic (PHC) for any health need.^20^ Before the BCCP was signed, it was assumed that the current intended coverage level for CBE screening (with 2 CBEs per year) was 5% of all eligible women. However, no record-keeping registry in South Africa tracks women who have undergone screening, making accurate estimation of past or current screening coverage challenging.

Because this policy was formulated in the absence of information on the efficacy and cost-effectiveness of scaling up CBE screening coverage in South Africa, we estimated the impact of scaling up CBE screening coverage on BC mortality and screen detection rates and the cost-effectiveness of the program.

## Methods

### Study Design

We developed a 2-part Microsoft Excel-based model that followed the breast health of cohorts of South Africa women. The first part, a decision tree, estimated the number of screen-detected or symptomatic cancers at baseline and scaled-up screening coverage levels. The second part, a state-transition model, tracked the population of women diagnosed with BC in the first part of the model. Using randomized clinical trial (RCT) data, we estimated the proportion of BCs downstaged through screening and followed the cohorts of women over their remaining lifetime. The model was used to simulate 2 scenarios in which women aged 40 to 69 years were targeted for screening regardless of symptoms, following BCCP guidelines. We analyzed screening scale-up coverage between 2023 and 2027, in accordance with the 2022 National Department of Health’s National Strategic Plan for the Prevention and Control of Non-Communicable Diseases (NCDs), which directs actions to be taken between 2022 and 2027 to address and reverse the threat posed by NCDs.^21^ The plan does not clearly articulate screening coverage targets but sets an overarching goal of reducing premature mortality from NCDs by one-third through prevention and treatment. It includes secondary prevention, which encompasses screening, diagnosis, and timely intervention to manage and control disease, reduce mortality, and minimize both disability and the overall burden of disease.

### Study Population

The model simulated the South Africa population accounting for births, background mortality, BC incidence, screening compliance, screen positivity rates, number of CBEs per annum, and CBE and mammography sensitivity. Women served by the public sector entered the model in 5-year age-banded cohorts (40-44, 45-49, 50-54, 55-59, 60-64, and 65-69 years) annually, allowing for the tracking of premature mortality and the aging of the population as BC incidence and all-cause mortality vary with age. For each age-banded cohort, the model allowed for the annual tracking of new incident BC cases and undetected BC cases from previous screening rounds.

The number of women in each age band was based on 2020 Statistics South Africa mid-year population estimates.^22^ Population growth was estimated at 1.30% per annum based on estimates from the same source in 2019 and 2020. Based on WHO estimates, it was assumed that the public sector served 80% of these women.^23^

### Model Structure

#### Part 1: Decision tree

**Figure 1:**
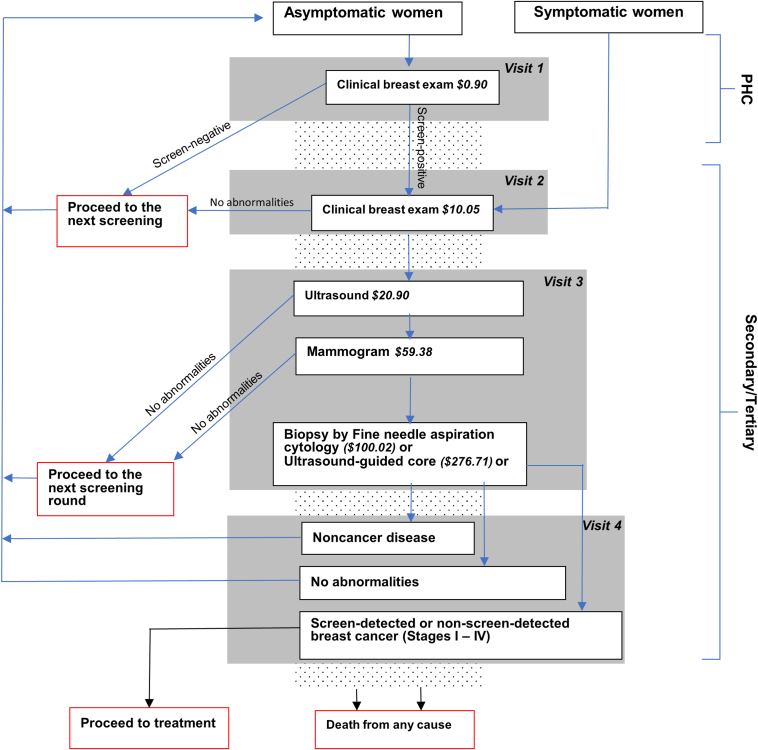
Screening and diagnosis cascade, including diagnosis in a specialist breast center, based on the clinical pathways provided in South Africa’s national policy. Women aged 40 to 69 enter the model as either asymptomatic or symptomatic patients. Asymptomatic women undergo their initial screening with a clinical CBE at a PHC, whereas symptomatic women receive their first screening with a CBE at a higher-level facility. Referrals from PHCs to higher-level facilities are made, and subsequent diagnostic steps are taken based on the abnormalities detected at each stage. CBE indicates clinical breast examination; PHC, primary healthcare clinic.

The decision tree tracked the movement of cohorts of women from screening to diagnosis, estimated the proportion of patients in different health states, and evaluated the screening and diagnostic costs associated with those states. Using the simulated cohort data, the model followed the clinical pathway provided in South Africa's national policy (Fig. 1 and Appendix Text 1 in Supplemental Materials found at https://doi.org/10.1016/j.vhri.2025.101127).

The proportion of incident BC cases detected via CBEs per screening round was based on the estimated CBE and mammography sensitivity.^24^^,^^25^ For simplicity, we assumed that the joint sensitivity of ultrasonography and all biopsies was 99% and designed the model to not allow for any loss to follow-ups at any point in the care continuum. Furthermore, patients were assumed to require only 1 diagnostic biopsy. Each scenario was implemented for 5 years to align with the National Department of Health’s Strategic Plan for the Prevention and Control of NCDs. Women re-entered the model every 6 months, corresponding with the intervention cycle length.

#### Part 2: State-transition model

**Figure 2:**
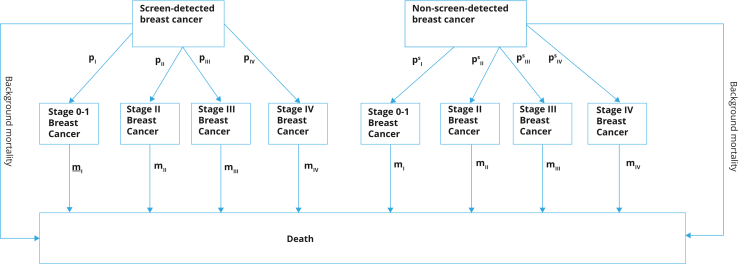
Clinical pathways of breast cancer treatment. Relationships between different health states, annual probabilities of being in each BC stage for “non-screen-detected BC” (psi) and “screen-detected BC” (pi), annual stage-specific BC fatality rates (mi), and background mortality. The BC stage was defined at the time of detection, and the assumption in the model was that there would be constant relapse or progression to stage 4 only, reflected in the disability weights. BC indicates breast cancer.

The structure of this state-transition cohort model (Fig. 2) was informed by the model from Groot et al^26^ and tracked women with screen-detected or non-screen-detected BC.^26^ Similar to an improved version of the Groot et al^26^ model,^27^ the assumption in our model was that women would relapse or progress to stage 4 only, which is reflected in the disability weights.

The cycle length of this part of the model was 1 year. The modeled cohorts were tracked for their remaining lifespan to determine the long-term impact of screening scale-up scenarios on costs and health. The midpoint age of each of the 6 age-banded cohorts was used to age women annually, with their BC incidence and background mortality rates (but not BC mortality) increasing with age. The history of each cohort was simulated based on the proportion of patients with screen-detected BC, age-specific incidence rates, and mortality rates.

### Comparators

Two screening scale-up scenarios were compared with baseline. Scenario 1 represented a slow approach (25% coverage by year 5, 5% annual increase), and scenario 2 was an aggressive approach (75% coverage by year 5, 17.5% annual increase). Detailed definitions of baseline and the 2 scale-up scenarios evaluated over 5 years (2022 to 2027):

#### Baseline scenario (no scale-up)

• Annual screening proportion: 5% annually, remaining level over the next 5 years.
• Proportion of eligible women intended to be screened in 2027: 5%.
• Estimated number of women screened in 2027: 234 188.

#### Scenario 1 (slow scale-up)

• Annual screening proportion: increases by 5% annually over the next 5 years.
• Proportion of eligible women intended to be screened in 2027: 25%.
• Estimated number of women screened in 2027: 1 170 938.

#### Scenario 2 (aggressive scale-up)

• Annual screening proportion: increases by 17.5% annually over the next 5 years.
• Proportion of eligible women intended to be screened in 2027: 75%.
• Estimated number of women screened in 2027: 3 512 815.

We did not carry out a full analysis of a no-screening baseline because we expected patient-requested screenings to continue even if provider-initiated screening stopped, given that women have the right to CBEs according to BCCP guidelines. However, we conducted an analysis to compare a no-screening scenario with baseline to better understand the economic efficiency of scaling up screening.

### Data Collection and Analysis: Downstaging, Screen-Positivity Rates, and Screening Uptake Rates

A literature search of RCTs determined the proportion of patients diagnosed with late-stage BC (stages 3-4) in the presence and absence of CBE screening (see Appendix Texts 2-4, Appendix Fig. 1, Appendix Tables 1-3 in Supplemental Materials found at https://doi.org/10.1016/j.vhri.2025.101127). A meta-analysis of data from 2 cluster RCTs^15^^,^^16^^,^^28^^,^^29^ that met our inclusion criteria indicated that CBE-based screening led to 9.7% of late-stage BCs in women aged 50 to 69 years and 10.10% in women under 50 years being downstaged (see Appendix Table 4 in Supplemental Materials found at https://doi.org/10.1016/j.vhri.2025.101127). Our model allowed for stage shifts by 1 category (4 to 3, 3 to 2, or 2 to 1) and explored a scenario in which some BCs shifted through 2 stages in a sensitivity analysis.

Key model parameters are summarized in Table 1.^15^^,^^16^^,^^23^^,^^24^^,^^28^, ^29^, ^30^, ^31^, ^32^, ^33^, ^34^, ^35^, ^36^

**Table 1:** Detailed specifications of the model parameters, mean values, and references for the data sources.

Parameter	Mean	Distribution and parameter estimates	Data source
2016 breast cancer incidence (constant over the 5 years)			^30^
Age band	Per 100 000 women	Beta distribution	
40-44	51.09	α = 940; β = 1 651 953	
45-49	70.3	α = 1083; β = 1 453 780	
50-54	85.36	α = 1134; β = 1 299 027	
55-59	102.34	α = 1198; β = 1 084 144	
60-64	127.04	α = 1098; β = 885 816	
65-69	131.58	α = 1006; β = 671 994	
Stage distribution of non-screen-detected breast cancers			^31^
Breast cancer stage	%	Beta distribution	
Stage 1	5.1%	α = 61; β = 1155	
Stage 2	41.2%	α = 491; β = 725	
Stage 3	44.7%	α = 533; β = 683	
Stage 4	9.0%	1-P_stage1_-P_stage2_-P_stage3_	
Case fatality rates of treated patients			^26^
Breast cancer stage	Annual fatality rate		
Stage 1	0.006		
Stage 2	0.042		
Stage 3	0.093		
Stage 4	0.275		
Sojourn times			^32^
Breast cancer stage	Years	Log-normal distribution	
Stage 1	4.885	Mean = 1.58608; SD = 0.21615	
Stage 2	2.487	Mean = 0.91094; SD = 0.10343	
Stage 3	0.702	Mean = -0.35398; SD = 0.28026	
Stage 4	0.674	Mean = -0.39527; SD = 0.27782	
Disability weights (corrected for relapse to stage 4)			^27^
Breast cancer stage	Disability weight		
Stage 1	0.068		
Stage 2	0.070		
Stage 3	0.072		
Stage 4	0.073		
Compliance with screening by 5-year age band			^16^^,^^28^
Age band	%		
40-44	69.19%	Beta distribution (α = 5618; β = 2502)	
45-49	71.02%	Beta distribution (α = 5790; β = 2363)	
50-54	71.24%	Beta distribution (α = 4906; β = 1981)	
55-59	71.77%	Beta distribution (α = 4315; β = 1698)	
60-64	71.89%	Beta distribution (α = 3813; β = 1491)	
65-69	69.13%	Beta distribution (α = 3542; β = 1574)	
Screen positivity rates			^16^^,^^28^
Age band	%		
40-44	7.91%	Beta distribution (α = 444; β = 5174)	
45-49	7.66%	Beta distribution (α = 443; β = 5347)	
50-54	5.96%	Beta distribution (α = 292; β = 4614)	
55-59	5.42%	Beta distribution (α = 234; β = 4081)	
60-64	4.58%	Beta distribution (α = 175; β = 3638)	
65-69	3.34%	Beta distribution (α = 118; β = 3406)	
Costs			
Screening and diagnostic costs			^33^
Procedure	2022 USD		
Ultrasound	$20.90		
Mammography	$59.38		
Biopsy by fine-needle aspiration cytology	$100.02		
Core biopsy: ultrasound guided	$276.71		
Core biopsy: stereotactic guided	$326.85		
Clinical breast exam (higher facility)	$10.05		
Follow-up diagnostic visit	$11.16		
Provider-initiated screening (CBE at PHC)	$0.90		∗
Treatment costs by breast cancer stage			^34^
Breast cancer stage	2022 USD		
Stage 1	$995		
Stage 2	$1101		
Stage 3	$1231		
Stage 4	$1230		
Accuracy of screening and diagnostic procedures			
CBE sensitivity	54.1%	Range: 40%-69%	^24^
Mammography sensitivity	87%		^35^
Biopsy method distribution			∗
Biopsy by fine-needle aspiration cytology	4%		
Core biopsy: ultrasound guided	84%		
Core biopsy: stereotactic guided	12%		
Other parameters			
Estimated downstaging (women aged <50 years)	10.10%	Beta distribution (α = 15; β = 112)	^15^^,^^16^^,^^29^
Estimated downstaging (women aged 50 years and older)	9.70%	Beta distribution (α = 13; β = 121)	^15^^,^^16^^,^^29^
Proportion of women seeking care in the public sector	80%		^23^
Annual inflation of future costs	6.7%		^36^

USD indicates US dollar.

∗ Breast cancer control policy budget impact analysis conducted by the Health Economics and Epidemiology Research Office (HE2RO).

### All-Cause Mortality and Life Expectancy Rates

Data on age-specific all-cause mortality rates were sourced from the WHO 2019 Life Tables for South African females.^37^ Life expectancy rates for calculating the years of life lost, defined as the number of deaths multiplied by the standard life expectancy at the age of death were also obtained from the WHO 2019 life tables.^37^

### Costs

Personnel costs of provider-initiated screening were based on our previous unpublished work conducted at the Health Economics and Epidemiology Research Office. Data on the costs of diagnostics included personnel, equipment, consumables, laboratory tests, and some overhead item costs and were sourced from a microcosting study that was conducted at a clinic in Johannesburg, South Africa, from 2013 to 2014.^33^ Data on treatment costs per episode of BC care (on average, a period of 10-12 months per patient) were obtained from a retrospective analysis of the cost of BC treatment at Groote Schuur Hospital in Cape Town, South Africa.^34^ The cost components included chemotherapy and support medicines, chemotherapy administration, consultations, laboratory tests, scans, and imaging. The estimated costs were for an episode of care, defined as the care provided 2 months before chemotherapy commenced, during chemotherapy and 6 months after the administration of the last chemotherapy dose. We assumed that noncancer lesions would not incur any treatment costs. Resource use and cost data were adjusted to the most recent salaries and prices and analyzed from the provider perspective.

The economic evaluation was carried out from the healthcare payer’s perspective and reported in 2022 US dollars (US $1 = ZAR 18.12, the average exchange rate in October 2022). Where necessary, costs were adjusted for inflation to 2022 ZAR using the South African Consumer Price index.^36^ A rate of 6.25%, the current reserve bank’s repo rate, was used to discount costs and health effects.^38^^,^^39^

### Modeling Outcomes

To measure intervention effectiveness, we determined the disability-adjusted life years (DALYs) averted, representing health loss from both fatal and nonfatal disease burdens. DALYs averted were calculated as the sum of the years of life lost, defined as the number of deaths multiplied by the standard life expectancy at the age of death, and years of life lived with disability, determined by multiplying the number of incident cases, duration, and disability weight for the condition.^40^, ^41^, ^42^, ^43^

The difference in lifetime costs between each analyzed scenario and baseline was divided by the difference in lifetime effects (DALYs) to estimate incremental cost-effectiveness ratios (ICERs). The ICERs were compared with the South African opportunity cost-derived cost-effectiveness threshold (OCCET) is $3015/DALY averted, which was derived by comparing gains in life expectancy from all healthcare to expenditures on all healthcare between 2000 and 2015.^44^ For each scenario, we estimated the cost per woman screened by dividing total screening and diagnostic costs by the number of women screened. We calculated 5-year screen detection rates per 1000 women screened by dividing the number of screen-detected BC cases over 5 years by the number of women screened, then multiplying by 1000.

### Sensitivity Analyses

We performed deterministic 1-way sensitivity analyses to assess the impact of varying the following parameters: screen positivity rates, estimated downstaging, treatment costs, CBE sensitivity, the proportion of BCs shifting 1 versus 2 stages, the proportion of women screened at baseline, the annual discount rate, and the cost of CBEs. For treatment cost variations, we used cost estimates from a BC control policy budget impact analysis conducted by the Health Economics and Epidemiology Research Office in 2017. These estimates, based on literature and South Africa’s Uniform Patient Fee Schedule, included costs for surgery, chemotherapy, radiation, and endocrine therapy. We also analyzed second-order uncertainty with a probabilistic sensitivity analysis using Monte Carlo simulation. We obtained 2000 estimates of DALYs averted and incremental costs by sampling from parameter distributions given in Table 1.^15^^,^^16^^,^^23^^,^^24^^,^^28^, ^29^, ^30^, ^31^, ^32^, ^33^, ^34^, ^35^, ^36^ This allowed an analysis of the impact of joint uncertainty in these parameters, which is represented on a cost-effectiveness plane and cost-effectiveness acceptability curves.

## Results

### Study Population and Screen Detection Rates

The number of women screened at baseline ranged from 223 570 in year 1 to 234 188 in year 5, and the number of women screened in scenarios 1 and 2 ranged from 223 570 to 1 170 938 and 3 512 815, respectively (see Appendix Table 5 in Supplemental Materials found at https://doi.org/10.1016/j.vhri.2025.101127). In scenario 2, the 5-year screen detection rate was 2.39 per 1000 women screened, whereas it was 2.08 per 1000 women screened in scenario 1. This suggests that rapid scale-up might result in a marginally lower detection rate over 5 years than slower, more gradual implementation.

### Proportion of Women Screened and the Impact of Screening on Mortality

At baseline, 5% of eligible women were targeted for screening, with actual screening uptake rates at 3.5% to 3.6%. In scenario 1, 25% were targeted, with 17.3% to 18% of women screened by year 5; in scenario 2, 75% were targeted, with 51.8% to 53.9% screened by year 5. Compared with baseline, scenario 1 reduced the years of life lost by 0.71%, whereas scenario 2 reduced the years of life lost by 2.25%. See Appendix Table 6 in Supplemental Materials found at https://doi.org/10.1016/j.vhri.2025.101127 for detailed data.

### Cost per Woman Screened

Our analysis of the cost per woman screened according to age band showed that costs decreased with age (see Appendix Table 7 in Supplemental Materials found at https://doi.org/10.1016/j.vhri.2025.101127). For baseline and scale-up scenarios, the average discounted costs per woman screened were very similar, at approximately $30. The costs of screening and diagnosis per screen-detected BC were $13 717, $12 653, and $14 476 for baseline and scenarios 1 and 2, respectively.

### Deterministic Results

Among women who were screen positive, the proportion of true positives after diagnostic workup was low at 3.9%, 4.2%, and 3.7% for baseline, scenario 1, and 2, respectively. All screen-positive cases had mammograms contributing to high total screening and diagnostic costs: $34 473 612 (baseline), $104 741 331(scenario 1), and $279 091 131 (scenario 2). Although treatment costs remained high across the scenarios, they decreased marginally because of downstaging, with total costs of $47 710 059 for the baseline, $46 932 942 for scenario 1, and $45 030 244 for scenario 2.

Compared with no screening, the current baseline screening program averts 1645 DALYs at an ICER of $20 341 per DALY averted. Implementation of scenario 1 would avert 3823 DALYs at a lower ICER of $17 776 per DALY averted, whereas scenario 2 would avert 12 165 DALYs at $19 552 per DALY averted. Detailed results are presented in Table 2.

**Table 2:** Deterministic results: Number screened; detection outcomes; screening, diagnostic, and treatment costs; discounted lifetime costs; effects; and ICERs of each scenario relative to baseline and no screening.

Scenario	No Screening	Baseline (40-69, 5% targeted annually)	Scenario 1 (40-69, slower scale-up)	Scenario 2 (40-69, aggressive scale-up)
Number of women eligible for screening in year 1	669 463			
Number of women screened over 5 years	0	1 144 834	3 460 445	9 249 475
Number of screen positives over 5 years∗	-	42 806	431 754	1 154 124
Number of screen positives that had a biopsy over 5 years	-	64 263	194 289	519 356
Number of true positives after diagnostic work up (over 5 years)	-	2513	8278	19 280
Stage 1 screen-detected	-	189	631	1267
Stage 2 screen-detected	-	1296	4271	9588
Stage 3 screen-detected	-	859	2820	7038
Stage 4 screen-detected	-	169	556	1387
Cost of all screening and diagnostics (over 5 years)	-	$34 473 612	$104 741 331	$279 091 131
Cost of diagnostic workup for those tested positive who turn out to be negative	-	$31 603 319	$95 822 071	$256 368 952
Cost of CBE for screen negatives	-	$1 960 772	$5 943 222	$15 899 349
Cost of diagnostic workup for true positives	-	$909 522	$2 976 037	$6 822 831
Cost of diagnostics + chemotherapy treatment (over 5 years)	$48 071 246	$47 710 059	$46 932 942	$45 030 244
Cost of diagnostics + chemotherapy treatment for stage 1	$2 213 480	$2 192 234	$2 145 953	$2 039 677
Cost of diagnostics + chemotherapy treatment for stage 2	$19 015 865	$18 993 208	$18 963 522	$18 795 005
Cost of diagnostics + chemotherapy treatment for stage 3	$22 344 788	$22 085 244	$21 512 107	$20 179 265
Cost of diagnostics + chemotherapy treatment for stage 4	$4 497 113	$4 439 374	$4 311 360	$4 016 297
Total discounted Cost (2022 USD)	$48 071 246	$81 535 350	$149 482 994	$319 392 198
Incremental cost (Compared with baseline)	−$33 464 104	-	$67 947 644	$237 856 848
Effectiveness (Total discounted DALYs)	521 074	519 429	515 606	507 264
Incremental effectiveness compared with baseline (DALYs averted)	−1645	-	3823	12 165
ICER (2022 USD per DALY averted) versus no screening		$20 341	$18 547	$19 646
ICER (2022 USD per DALY averted) versus baseline	$20 341		$17 776	$19 552

DALY indicates disability-adjusted life year; ICER, incremental cost-effectiveness ratio; USD, US dollar.

∗ 100% of screen-positive women have mammograms.

### Probabilistic Sensitivity Analysis

**Figure 3:**
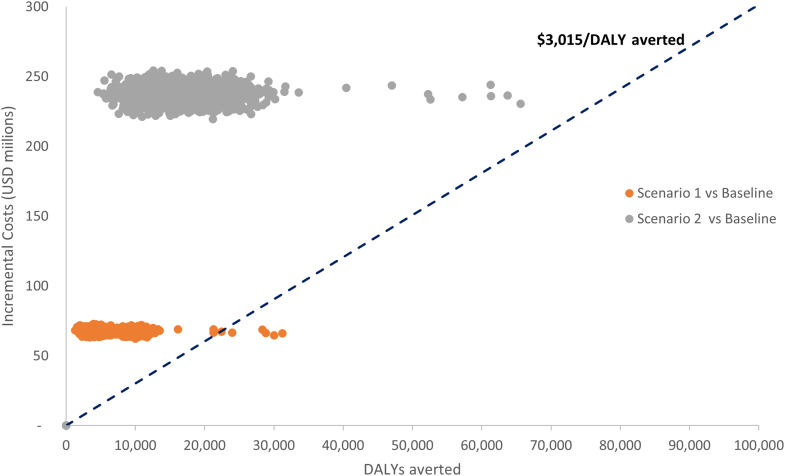
Population-level cost-effectiveness plane: displaying aggregate outcomes across 2000 Simulations. DALY indicates disability-adjusted life year; USD, US dollar.

The range of 2000 simulated ICERs over the baseline (Fig. 3) was $2118 to $51 778 and $3513 to $52 088/DALY averted for scenarios 1 and 2, respectively. All observations were in the northeast quadrant of the cost-effectiveness plane, in which the intervention was both more effective and more costly than baseline.

### 1-Way Sensitivity Analyses

**Figure 4:**
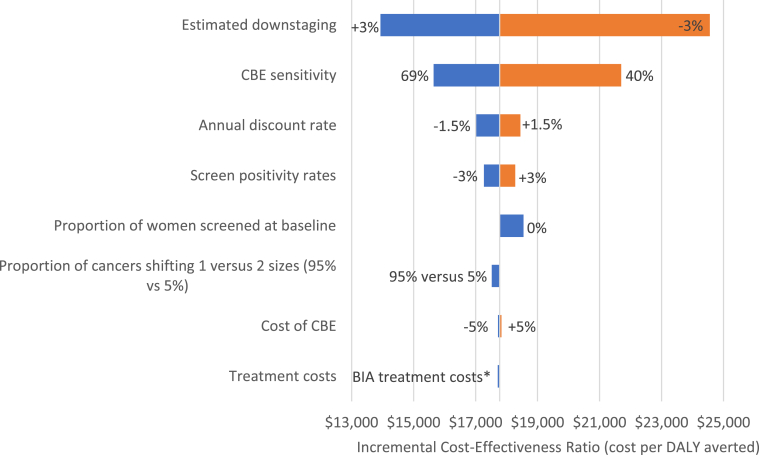
Tornado plot showing the results of a 1-way sensitivity analysis of relevant model parameters. The horizontal axis represents the incremental cost-effectiveness ratio (ICER) of scenario 1 compared with the baseline, which is truncated at $17 776 and is the deterministic ICER of scenario 1. The uncertainty in the parameter with the largest bar at the top of the chart (the screen positivity rates) has the maximum impact on the result, with each successive lower bar representing parameters with progressively lesser impacts. ∗BIA treatment cost estimates from the breast cancer control policy budget impact analysis conducted by the Health Economics and Epidemiology Research Office (HE2RO) in 2017. These estimates, based on literature and South Africa’s Uniform Patient Fee Schedule, include costs for surgery, chemotherapy, radiation, and endocrine therapy. CBE indicates clinical breast examination; BIA, budget impact analysis; DALY, disability-adjusted life year.

Model outcomes were most sensitive to our assumption regarding downstaging rates, followed by the CBE sensitivity (Fig. 4). Our model was the least sensitive to the costs of CBEs and treatment costs.

### Cost-effectiveness Acceptability Curve

The cost-effectiveness acceptability curve (see Fig. 2 in Supplemental Materials found at https://doi.org/10.1016/j.vhri.2025.101127) illustrates the proportion of probabilistic sensitivity analysis iterations with the highest net monetary benefit for each scenario, indicating the most cost-effective scenario at different cost-effectiveness thresholds. The net monetary benefit was calculated as follows: the incremental benefit × cost-effectiveness threshold − incremental cost. At an OCCET of $3015/DALY averted, the baseline scenario was 100% more likely to be cost-effective than the alternative scenarios.

## Discussion

To our knowledge, this study is the first to analyze the impact of increasing the coverage level of BC screening in South African women aged 40 to 69 years over 5 years, whether gradually (scenario 1: 25% coverage by year 5, 5% annual increase) or aggressively (scenario 2: 75% coverage by year 5, 17.5% annual increase). Scenario 1 averted 3823 DALYs, whereas scenario 2 averted 12 165 DALYs at $17 776 and $19 552/DALY averted. At current 5% screening rates, the program averts DALYs at a cost of $20 341/DALY averted compared with no screening, with economic efficiency improving under scenario 1. We demonstrated that scaling up BC screening using CBEs is effective in saving life years but not cost-effective under an OCCET of $3015/DALY averted. Scenario 2 had slightly lower 5-year BC screen detection rates than scenario 1. Costs per woman screened decreased with age and varied between scenarios. Scenario 1 reduced BC mortality by 0.7% and scenario 2 by 2.3%. Model outcomes were sensitive to the success of downstaging, CBE sensitivity, and screening frequency.

Although CBE scale-up is not cost-effective under the OCCET, this metric alone does not dictate healthcare decisions in South Africa. There is some evidence that the South African government’s willingness to pay for cancer care is higher than indicated by the OCCET in the sense that it is funding other cancer interventions with slightly higher ICERs ($5381 for capecitabine and oxaliplatin treatment for colon cancer).^45^ Consequently, the choice of the most appropriate screening policy for South Africa will depend largely on government’s willingness to pay, considering other competing health priorities.

To date, no other cost-effectiveness analyses of the South Africa BC screening policy using data from RCTs exist. Our findings support a previous analysis of the Moroccan BC screening policy, which estimated that screening women between 45 and 69 years of age every 2 years using CBEs at a screening coverage rate of 32% was not cost-effective compared with no screening, with an estimated ICER of $32 388 per life year saved.^46^ Consequently, our study holds broad significance for other LMICs evaluating similar BC screening strategies. Another key strength of our study is the precise parameterization of our model using data from relevant settings, specifically South Africa and similar settings.

Our study has certain limitations. Some key parameters in our model were based on 2 RCTs conducted in India, where BC incidence rates are lower than those in South Africa. In 2019, South Africa had an estimated age-standardized incidence rate (ASIR) of 33.95 cases (CI 33.29-34.63) per 100 000 women versus 25.8 cases in India.^30^^,^^47^ However, the 2 Indian cities included in these 2 RCTs (Trivandrum and Mumbai) had some of the highest BC ASIRs comparable to South Africa BC ASIRs.^15^^,^^16^ Additionally, India faces challenges in healthcare access, screening infrastructure, and disparities in cancer treatment similar to those in South Africa.

In the absence of country-wide data, data from single provinces were used for certain parameters: (1) data on the stage distribution of non-screen-detected BC cases were sourced from Eastern Cape, and (2) data on BC treatment costs were sourced from a study carried out in Johannesburg. These parameters may not represent country-level estimates. However, a large percentage of the input costs used in these studies included drug, laboratory, and diagnostic tests set at the national level.

The model assumed that 100% of all BC patients would have access to and take up treatment; however, 1 of the Indian RCTs included in our study revealed that only 92% of women with screen-detected BC sought treatment services.^29^ Therefore, allowing for reduced treatment uptake rates would not change the overall conclusions but rather increase the ICERs, making all scenarios even less cost-effective.

Given the absence of South African-specific data on the impact of CBE screening on BC mortality, the model did not make any direct assumptions on the effectiveness of screening in reducing mortality rates. Instead, it relied on estimates of downstaging as a proxy measure for BC mortality. Downstaging serves as a valid proxy for mortality, and the estimated impact on mortality corresponds with definitive findings of the Trivandrum RCT after a 14-year follow-up, in which CBEs failed to demonstrate any mortality benefit.^16^

Owing to a lack of published data on comprehensive BC care costs—including surgery, radiation therapy, trastuzumab and hormone therapy, follow-up care, palliative care, and reconstructive surgery—our primary analysis incorporated only chemotherapy costs. However, when we conducted a sensitivity analysis using more comprehensive treatment cost estimates from our previous work, we found only marginal impacts on the ICERs. This suggests that our findings remain robust despite this data limitation.

The costs of capacity building, including training service providers, promoting CBEs via media, and developing structures to address the increased BC screening rates, were not included. Including these additional treatment-related costs would not be expected to affect our findings because they would affect the baseline and the compared scenarios uniformly. However, incorporating screening-related costs specifically would increase the ICERs. Future research should aim to include these comprehensive cost elements to provide a more detailed economic evaluation of BC screening programs in LMICs.

Our analysis assumes uniform implementation and access to CBE screening across populations, but this does not reflect the reality of South Africa's healthcare landscape.^48^ Significant disparities exist in healthcare access and may affect the distribution of benefits from CBE screening. For example, populations in rural areas or with lower socioeconomic status may experience challenges in accessing healthcare services, including screening, because of limited infrastructure, financial barriers, and lack of healthcare professionals.^49^ For CBE screening to be equitable and effective, implementation strategies would need to explicitly address these structural barriers through targeted outreach programs, mobile screening services, and broader health system strengthening initiatives.

The effectiveness of CBE as a screening tool is limited by its lower sensitivity compared with imaging-based methods, increasing the risk of missed diagnoses or false positives. Reported sensitivity for CBE ranges from of 40% to 69%, much lower than 77% to 79% for mammography.^24^ Studies show that untrained practitioners or those without standardized protocols achieve much lower sensitivity rates (28-36%) compared with practitioners in structured screening programs.^24^^,^^50^1This performance gap highlights the method’s dependency on practitioner skill, necessitating adequate training.

Screening initiatives may increase demand for BC diagnostic and treatment centers. Therefore, establishing a consensus on minimum care standards for each stage of BC is essential before implementing or scaling up an organized screening program. This includes assessing whether early diagnosis, focused on detecting BC in symptomatic patients as early as possible, is a more cost-effective approach for achieving early detection in South African.

Scaling up CBE is effective in saving life years and slightly reduces treatment costs. Although economic efficiency improves as coverage expands, implementing CBE scale-up requires investment, and decision makers must weigh this against other healthcare priorities, including interventions for other diseases. Given the OCCET threshold of $3015 per DALY averted, alternative interventions may also be considered. However, decisions on implementation are not based solely on cost-effectiveness.

## Conclusions

Using a cohort model and trial downstaging data, we found that scaling up BC screening using CBEs in South Africa is effective in saving life years but not cost-effective under an OCCET of $3015/DALY averted. However, decisions regarding the best screening policy for South Africa are not solely based on cost-effectiveness. They also involve careful consideration of budgetary constraints and competing healthcare priorities. Additionally, screening initiatives may increase demand for diagnostic and treatment centers. Therefore, establishing minimum care standards and evaluating the most cost-effective early detection methods is essential before scaling up the BC screening program in South Africa.

## Article and Author Information

**Author Contributions:** *Concept and design:* Masuku, Mandrik, Brennan

*Supervision:* Mandrik, Brennan

*Model construction:* Masuku

*Model validation:* Mandrik, Mdege

*Review of results:* Mandrik, Mdege, Mishra, Lince-Deroche, Brennan

*Drafting of the manuscript:* Masuku

*Review and editing of the manuscript:* Masuku, Mandrik, Mdege, Mishra, Muwonge, Meyer-Rath, Lince-Deroche, Brennan

**Funding/Support:** This work was supported by the 10.13039/501100000272National Institute for Health Research (NIHR) (using the UK’s Official Development Assistance Funding) and Wellcome (218334/Z/19/Z) under the NIHR-Wellcome Partnership for 10.13039/100006090Global Health Research to S.D.M.

**Role of the Funder/Sponsor:** The funder had no role in the design and conduct of the study; collection, management, analysis, and interpretation of the data; preparation, review, or approval of the manuscript; or decision to submit the manuscript for publication.

**Data Availability:** Data used as input for the model can be requested from the primary sources, as specified in Table 1^15^^,^^16^^,^^23^^,^^24^^,^^28^^,^^29^^,^^30^, ^31^, ^32^, ^33^, ^34^, ^35^, ^36^ and Appendix Table 2 in Supplemental Materials found at https://doi.org/10.1016/j.vhri.2025.101127.

**Ethical Approval:** No ethics approval was needed.

**Disclaimer:** Where authors are identified as personnel of the International Agency for Research on Cancer/World Health Organization, the authors alone are responsible for the views expressed in this article, and they do not necessarily represent the decisions, policy or views of the International Agency for Research on Cancer/World Health Organization.

For the purpose of Open Access, the author has applied a CC BY public copyright license to any Author Accepted Manuscript version arising from this submission.

## Author Disclosures

Author disclosure forms can be accessed below in the Supplemental Material section. The views expressed are those of the authors and not necessarily those of Wellcome, the NIHR or the UK Department of Health and Social Care.

## Supplemental Material

Author Disclosures
