# Supplementary material for: Breast Cancer Screening Using Clinical Breast Examination: A Cost-Effectiveness Analysis for South Africa
Source: Value Health Reg Issues. 2025 Sep;49:None. doi: 10.1016/j.vhri.2025.101127 (PMC12411605; doi:10.1016/j.vhri.2025.101127)
Supplement: Author Disclosures [file mmc1.pdf]

Alan Brennan

Discloser Identifier: 55019814

Disclosure Purpose: VIHRI-CEEWAA-2024-0288

Employment Information: Currently Employed

Summary of Interests

Company or Organization

Entity	Type	Relevant to this Disclosure
NIHR	Grant / Contract	
University of Sheffield	Employment Current Employment	
**Title:** Professor of Health Economics & Decision Modelling		
Wellcome Trust	Grant / Contract	Yes

Additional Questions

1. **Please select which of the following apply to each relationship or activity:**
   a. **Employment** University of Sheffield

      The relationship is in direct support of the work reported in the manuscript anytime from when the work was conceived

   b. **Grant / Contract** NIHR

      Neither

   c. **Grant / Contract** Wellcome Trust

      The relationship is in direct support of the work reported in the manuscript anytime from when the work was conceived

2. **I confirm I have disclosed all direct support for the present manuscript (e.g. funding, provision of study materials, medical writing, article processing charges, etc.) There is no time limit for this item.**

   Yes

3. **Please indicate below whether in the past 36 months you have had any of the following interests that are topically related to the work reported in the manuscript.**
   a. **Employment (If you need to add an interest, please scroll to the top of the page, click "add interest" and select "Employment")**

      Yes, as disclosed above

   b. **Grants or contracts for research (If you need to add an interest, please scroll to the top of the page, click "add interest" and select "Grant/Contract")**

      Yes, as disclosed above

   c. **Payment for consulting (If you need to add an interest, please scroll to the top of the page, click "add interest" and select "Independent Contractor")**

      No, I have no relevant interests of this type

d. **Payments or honoraria for lectures, presentations, speakers bureaus, or educational events (If you need to add an interest, please scroll to the top of the page, click "add interest" and select "Independent Contractor" and include the correct information under "Consultant")**

   No, I have no relevant interests of this type

e. **Payment for service on an advisory board (If you need to add an interest, please scroll to the top of the page, click "add interest" and select “Independent Contractor,” and choose “Other”)**

   No, I have no relevant interests of this type

f. **Payment for participation Data and safety monitoring board (If you need to add an interest, please scroll to the top of the page, click "add interest" and select “Independent Contractor”)**

   No, I have no relevant interests of this type

g. **Expert witness testimony (If you need to add an interest, please scroll to the top of the page, click "add interest" and select “Independent Contractor”)**

   No, I have no relevant interests of this type

h. **Royalties from Patents, Trademarks, Copyrights or other intellectual property (If you need to add an interest, please scroll to the top of the page, click "add interest" and select the appropriate interest type)**

   No, I have no relevant interests of this type

i. **Patents planned, issued, or pending, whether or not you receive royalties (If you need to add an interest, please scroll to the top of the page, click "add interest" and select “Patents”)**

   No, I have no relevant interests of this type

j. **Fiduciary Officer or Other Board Membership (If you need to add an interest, please scroll to the top of the page, click "add interest" and select “Fiduciary Officer”)**

   No, I have no relevant interests of this type

k. **Stock or stock options (If you need to add an interest, please scroll to the top of the page, click "add interest" and select the appropriate interest type)**

   No, I have no relevant interests of this type

l. **Support for attending meetings or other travel (If you need to add an interest, please scroll to the top of the page, click "add interest" and select “Travel”)**

   No, I have no relevant interests of this type

4. **Was any individual paid to provide professional writing assistance with this manuscript?**

   No.

5. **Have you or your institution received equipment, materials, drugs, or services in direct support of the work in the manuscript (without time limit) not disclosed above?**

   No.

6. **In the past 36 months, have you received equipment, materials, drugs, medical writing, gifts or other services from for-profit or not-for-profit third parties whose interests may be affected by the content of the manuscript not disclosed above?**

   No.

7. **Are there other financial or non-financial interests that readers could perceive to have influenced, or that give the appearance of potentially influencing, what you wrote in the submitted work not disclosed above.**

   No.

Certification

I certify that I have answered every question and the information provided in this disclosure is complete and accurate.

Naomi Lince-Deroche

Discloser Identifier: 126251262

Disclosure Purpose: VIHRI-CEEWAA-2024-0288

Employment Information: Currently Employed

Summary of Interests

I do not have any interests to disclose at this time.

Additional Questions

1. **Please select which of the following apply to each relationship or activity:**

   You are not disclosing any interests to this organization.

2. **I confirm I have disclosed all direct support for the present manuscript (e.g. funding, provision of study materials, medical writing, article processing charges, etc.) There is no time limit for this item.**

   Yes

3. **Please indicate below whether in the past 36 months you have had any of the following interests that are topically related to the work reported in the manuscript.**

   a. **Employment (If you need to add an interest, please scroll to the top of the page, click "add interest" and select “Employment”)**

      No, I have no relevant interests of this type

   b. **Grants or contracts for research (If you need to add an interest, please scroll to the top of the page, click "add interest" and select “Grant/Contract”)**

      No, I have no relevant interests of this type

   c. **Payment for consulting (If you need to add an interest, please scroll to the top of the page, click "add interest" and select “Independent Contractor”)**

      No, I have no relevant interests of this type

   d. **Payments or honoraria for lectures, presentations, speakers bureaus, or educational events (If you need to add an interest, please scroll to the top of the page, click "add interest" and select "Independent Contractor" and include the correct information under "Consultant")**

      No, I have no relevant interests of this type

   e. **Payment for service on an advisory board (If you need to add an interest, please scroll to the top of the page, click "add interest" and select “Independent Contractor,” and choose “Other”)**

      No, I have no relevant interests of this type

   f. **Payment for participation Data and safety monitoring board (If you need to add an interest, please scroll to the top of the page, click "add interest" and select “Independent Contractor”)**

      No, I have no relevant interests of this type

   g. **Expert witness testimony (If you need to add an interest, please scroll to the top of the page, click "add interest" and select “Independent Contractor”)**

      No, I have no relevant interests of this type

   h. **Royalties from Patents, Trademarks, Copyrights or other intellectual property (If you need to add an interest, please scroll to the top of the page, click "add interest" and select the appropriate interest type)**

No, I have no relevant interests of this type

i. **Patents planned, issued, or pending, whether or not you receive royalties (If you need to add an interest, please scroll to the top of the page, click "add interest" and select “Patents”)**

No, I have no relevant interests of this type

j. **Fiduciary Officer or Other Board Membership (If you need to add an interest, please scroll to the top of the page, click "add interest" and select “Fiduciary Officer”)**

No, I have no relevant interests of this type

k. **Stock or stock options (If you need to add an interest, please scroll to the top of the page, click "add interest" and select the appropriate interest type)**

No, I have no relevant interests of this type

l. **Support for attending meetings or other travel (If you need to add an interest, please scroll to the top of the page, click "add interest" and select “Travel”)**

No, I have no relevant interests of this type

4. **Was any individual paid to provide professional writing assistance with this manuscript?**

No.

5. **Have you or your institution received equipment, materials, drugs, or services in direct support of the work in the manuscript (without time limit) not disclosed above?**

No.

6. **In the past 36 months, have you received equipment, materials, drugs, medical writing, gifts or other services from for-profit or not-for-profit third parties whose interests may be affected by the content of the manuscript not disclosed above?**

No.

7. **Are there other financial or non-financial interests that readers could perceive to have influenced, or that give the appearance of potentially influencing, what you wrote in the submitted work not disclosed above.**

No.

Certification

I certify that I have answered every question and the information provided in this disclosure is complete and accurate.

Olena Mandrik

**Discloser Identifier:**	95703288	**Disclosure Purpose:**	VIHRI-CEEWAA-2024-0288	**Employment Information:**	Currently Employed

Summary of Interests

Company or Organization

Entity	Type	Relevant to this Disclosure
London School of Economics and Political Science	Consultant	Yes
**Category:** Consultant		
World Bank Group	Consultant Current Employment	Yes
**Category:** Consultant		

Additional Questions

1. **Please select which of the following apply to each relationship or activity:**
   a. **Other Professional Activities - Consultant** London School of Economics and Political Science

      The relationship is outside the work reported in the manuscript but topically related and within the past 36 months

   b. **Other Professional Activities - Consultant** World Bank Group

      The relationship is outside the work reported in the manuscript but topically related and within the past 36 months

2. **I confirm I have disclosed all direct support for the present manuscript (e.g. funding, provision of study materials, medical writing, article processing charges, etc.) There is no time limit for this item.**

   Yes

3. **Please indicate below whether in the past 36 months you have had any of the following interests that are topically related to the work reported in the manuscript.**
   a. **Employment (If you need to add an interest, please scroll to the top of the page, click "add interest" and select "Employment")**

      Yes, as disclosed above

   b. **Grants or contracts for research (If you need to add an interest, please scroll to the top of the page, click "add interest" and select "Grant/Contract")**

      No, I have no relevant interests of this type

   c. **Payment for consulting (If you need to add an interest, please scroll to the top of the page, click "add interest" and select "Independent Contractor")**

      Yes, as disclosed above

   d. **Payments or honoraria for lectures, presentations, speakers bureaus, or educational events (If you need to add an interest, please scroll to the top of the page, click "add interest" and select "Independent Contractor" and include the correct information under "Consultant")**

Yes, as disclosed above

e. **Payment for service on an advisory board (If you need to add an interest, please scroll to the top of the page, click "add interest" and select “Independent Contractor,” and choose “Other”)**

No, I have no relevant interests of this type

f. **Payment for participation Data and safety monitoring board (If you need to add an interest, please scroll to the top of the page, click "add interest" and select “Independent Contractor”)**

No, I have no relevant interests of this type

g. **Expert witness testimony (If you need to add an interest, please scroll to the top of the page, click "add interest" and select “Independent Contractor”)**

No, I have no relevant interests of this type

h. **Royalties from Patents, Trademarks, Copyrights or other intellectual property (If you need to add an interest, please scroll to the top of the page, click "add interest" and select the appropriate interest type)**

No, I have no relevant interests of this type

i. **Patents planned, issued, or pending, whether or not you receive royalties (If you need to add an interest, please scroll to the top of the page, click "add interest" and select “Patents”)**

No, I have no relevant interests of this type

j. **Fiduciary Officer or Other Board Membership (If you need to add an interest, please scroll to the top of the page, click "add interest" and select “Fiduciary Officer”)**

No, I have no relevant interests of this type

k. **Stock or stock options (If you need to add an interest, please scroll to the top of the page, click "add interest" and select the appropriate interest type)**

No, I have no relevant interests of this type

l. **Support for attending meetings or other travel (If you need to add an interest, please scroll to the top of the page, click "add interest" and select “Travel”)**

Yes, as disclosed above

4. **Was any individual paid to provide professional writing assistance with this manuscript?**

No.

5. **Have you or your institution received equipment, materials, drugs, or services in direct support of the work in the manuscript (without time limit) not disclosed above?**

No.

6. **In the past 36 months, have you received equipment, materials, drugs, medical writing, gifts or other services from for-profit or not-for-profit third parties whose interests may be affected by the content of the manuscript not disclosed above?**

No.

7. **Are there other financial or non-financial interests that readers could perceive to have influenced, or that give the appearance of potentially influencing, what you wrote in the submitted work not disclosed above.**

No.

Certification

I certify that I have answered every question and the information provided in this disclosure is complete and accurate.

Sithabiso Masuku

Discloser Identifier: 126249518

Disclosure Purpose: VIHRI-CEEWAA-2024-0288

Employment Information: Currently Employed

Summary of Interests

Company or Organization

Entity	Type	Relevant to this Disclosure
Breast Cancer Research Foundation	Other	Yes
**Category:** Other		
Health Economics and Epidemiology Research Office	Other	
**Category:** Other		
University of Sheffield	Other Current Employment	Yes
**Category:** Other		
Wellcome Trust	Grant / Contract	Yes

Additional Questions

1. **Please select which of the following apply to each relationship or activity:**
   a. **Other Professional Activities - Other** Breast Cancer Research Foundation

      The relationship is in direct support of the work reported in the manuscript anytime from when the work was conceived

   b. **Other Professional Activities - Other** Health Economics and Epidemiology Research Office

      The relationship is in direct support of the work reported in the manuscript anytime from when the work was conceived

   c. **Other Professional Activities - Other** University of Sheffield

      The relationship is in direct support of the work reported in the manuscript anytime from when the work was conceived

   d. **Grant / Contract** Wellcome Trust

      The relationship is in direct support of the work reported in the manuscript anytime from when the work was conceived

2. **I confirm I have disclosed all direct support for the present manuscript (e.g. funding, provision of study materials, medical writing, article processing charges, etc.) There is no time limit for this item.**

   Yes

3. **Please indicate below whether in the past 36 months you have had any of the following interests that are topically related to the work reported in the manuscript.**
   a. **Employment (If you need to add an interest, please scroll to the top of the page, click "add interest" and select “Employment”)**

Yes, as disclosed above

b. **Grants or contracts for research (If you need to add an interest, please scroll to the top of the page, click "add interest" and select "Grant/Contract")**

   Yes, as disclosed above

c. **Payment for consulting (If you need to add an interest, please scroll to the top of the page, click "add interest" and select "Independent Contractor")**

   No, I have no relevant interests of this type

d. **Payments or honoraria for lectures, presentations, speakers bureaus, or educational events (If you need to add an interest, please scroll to the top of the page, click "add interest" and select "Independent Contractor" and include the correct information under "Consultant")**

   No, I have no relevant interests of this type

e. **Payment for service on an advisory board (If you need to add an interest, please scroll to the top of the page, click "add interest" and select "Independent Contractor," and choose "Other")**

   No, I have no relevant interests of this type

f. **Payment for participation Data and safety monitoring board (If you need to add an interest, please scroll to the top of the page, click "add interest" and select "Independent Contractor")**

   No, I have no relevant interests of this type

g. **Expert witness testimony (If you need to add an interest, please scroll to the top of the page, click "add interest" and select "Independent Contractor")**

   No, I have no relevant interests of this type

h. **Royalties from Patents, Trademarks, Copyrights or other intellectual property (If you need to add an interest, please scroll to the top of the page, click "add interest" and select the appropriate interest type)**

   No, I have no relevant interests of this type

i. **Patents planned, issued, or pending, whether or not you receive royalties (If you need to add an interest, please scroll to the top of the page, click "add interest" and select "Patents")**

   No, I have no relevant interests of this type

j. **Fiduciary Officer or Other Board Membership (If you need to add an interest, please scroll to the top of the page, click "add interest" and select "Fiduciary Officer")**

   No, I have no relevant interests of this type

k. **Stock or stock options (If you need to add an interest, please scroll to the top of the page, click "add interest" and select the appropriate interest type)**

   No, I have no relevant interests of this type

l. **Support for attending meetings or other travel (If you need to add an interest, please scroll to the top of the page, click "add interest" and select "Travel")**

   No, I have no relevant interests of this type

4. **Was any individual paid to provide professional writing assistance with this manuscript?**

   Yes.

   a. **Please identify the individual or entity that provided the payment for the writing assistance and any other relevant details.**

      The manuscript underwent professional editing by the Nature editing team. As a fellow of the inaugural Nature x Breast Cancer Research Foundation (BCRF) Rising Scholars program, I received editing credits which were used for this purpose. The editing credits would h

5. **Have you or your institution received equipment, materials, drugs, or services in direct support of the work in the manuscript (without time limit) not disclosed above?**

   No.

6. **In the past 36 months, have you received equipment, materials, drugs, medical writing, gifts or other services from for-profit or not-for-profit third parties whose interests may be affected by the content of the manuscript not disclosed above?**

   No.

7. **Are there other financial or non-financial interests that readers could perceive to have influenced, or that give the appearance of potentially influencing, what you wrote in the submitted work not disclosed above.**

   No.

Certification

I certify that I have answered every question and the information provided in this disclosure is complete and accurate.

Convey Global Disclosure System | AAMC

Noreen Mdege

Discloser Identifier: 126251127

Disclosure Purpose: VIHRI-CEEWAA-2024-0288

Employment Information: Currently Employed

Summary of Interests

I do not have any interests to disclose at this time.

Additional Questions

1. **Please select which of the following apply to each relationship or activity:**

   You are not disclosing any interests to this organization.

2. **I confirm I have disclosed all direct support for the present manuscript (e.g. funding, provision of study materials, medical writing, article processing charges, etc.) There is no time limit for this item.**

   Yes

3. **Please indicate below whether in the past 36 months you have had any of the following interests that are topically related to the work reported in the manuscript.**

   a. **Employment (If you need to add an interest, please scroll to the top of the page, click "add interest" and select “Employment”)**

      No, I have no relevant interests of this type

   b. **Grants or contracts for research (If you need to add an interest, please scroll to the top of the page, click "add interest" and select “Grant/Contract”)**

      No, I have no relevant interests of this type

   c. **Payment for consulting (If you need to add an interest, please scroll to the top of the page, click "add interest" and select “Independent Contractor”)**

      No, I have no relevant interests of this type

   d. **Payments or honoraria for lectures, presentations, speakers bureaus, or educational events (If you need to add an interest, please scroll to the top of the page, click "add interest" and select "Independent Contractor" and include the correct information under "Consultant")**

      No, I have no relevant interests of this type

   e. **Payment for service on an advisory board (If you need to add an interest, please scroll to the top of the page, click "add interest" and select “Independent Contractor,” and choose “Other”)**

      No, I have no relevant interests of this type

   f. **Payment for participation Data and safety monitoring board (If you need to add an interest, please scroll to the top of the page, click "add interest" and select “Independent Contractor”)**

      No, I have no relevant interests of this type

   g. **Expert witness testimony (If you need to add an interest, please scroll to the top of the page, click "add interest" and select “Independent Contractor”)**

      No, I have no relevant interests of this type

   h. **Royalties from Patents, Trademarks, Copyrights or other intellectual property (If you need to add an interest, please scroll to the top of the page, click "add interest" and select the appropriate interest type)**

No, I have no relevant interests of this type

i. **Patents planned, issued, or pending, whether or not you receive royalties (If you need to add an interest, please scroll to the top of the page, click "add interest" and select “Patents”)**

No, I have no relevant interests of this type

j. **Fiduciary Officer or Other Board Membership (If you need to add an interest, please scroll to the top of the page, click "add interest" and select “Fiduciary Officer”)**

No, I have no relevant interests of this type

k. **Stock or stock options (If you need to add an interest, please scroll to the top of the page, click "add interest" and select the appropriate interest type)**

No, I have no relevant interests of this type

l. **Support for attending meetings or other travel (If you need to add an interest, please scroll to the top of the page, click "add interest" and select “Travel”)**

No, I have no relevant interests of this type

4. **Was any individual paid to provide professional writing assistance with this manuscript?**

No.

5. **Have you or your institution received equipment, materials, drugs, or services in direct support of the work in the manuscript (without time limit) not disclosed above?**

No.

6. **In the past 36 months, have you received equipment, materials, drugs, medical writing, gifts or other services from for-profit or not-for-profit third parties whose interests may be affected by the content of the manuscript not disclosed above?**

No.

7. **Are there other financial or non-financial interests that readers could perceive to have influenced, or that give the appearance of potentially influencing, what you wrote in the submitted work not disclosed above.**

No.

Certification

I certify that I have answered every question and the information provided in this disclosure is complete and accurate.

Gesine Meyer-Rath

Discloser Identifier: 90732962

Disclosure Purpose: VIHRI-CEEWAA-2024-0288

Employment Information: Currently Employed

Summary of Interests

I do not have any interests to disclose at this time.

Additional Questions

1. **Please select which of the following apply to each relationship or activity:**

   You are not disclosing any interests to this organization.

2. **I confirm I have disclosed all direct support for the present manuscript (e.g. funding, provision of study materials, medical writing, article processing charges, etc.) There is no time limit for this item.**

   Yes

3. **Please indicate below whether in the past 36 months you have had any of the following interests that are topically related to the work reported in the manuscript.**

   a. **Employment (If you need to add an interest, please scroll to the top of the page, click "add interest" and select “Employment”)**

      No, I have no relevant interests of this type

   b. **Grants or contracts for research (If you need to add an interest, please scroll to the top of the page, click "add interest" and select “Grant/Contract”)**

      No, I have no relevant interests of this type

   c. **Payment for consulting (If you need to add an interest, please scroll to the top of the page, click "add interest" and select “Independent Contractor”)**

      No, I have no relevant interests of this type

   d. **Payments or honoraria for lectures, presentations, speakers bureaus, or educational events (If you need to add an interest, please scroll to the top of the page, click "add interest" and select "Independent Contractor" and include the correct information under "Consultant")**

      No, I have no relevant interests of this type

   e. **Payment for service on an advisory board (If you need to add an interest, please scroll to the top of the page, click "add interest" and select “Independent Contractor,” and choose “Other”)**

      No, I have no relevant interests of this type

   f. **Payment for participation Data and safety monitoring board (If you need to add an interest, please scroll to the top of the page, click "add interest" and select “Independent Contractor”)**

      No, I have no relevant interests of this type

   g. **Expert witness testimony (If you need to add an interest, please scroll to the top of the page, click "add interest" and select “Independent Contractor”)**

      No, I have no relevant interests of this type

   h. **Royalties from Patents, Trademarks, Copyrights or other intellectual property (If you need to add an interest, please scroll to the top of the page, click "add interest" and select the appropriate interest type)**

No, I have no relevant interests of this type

i. **Patents planned, issued, or pending, whether or not you receive royalties (If you need to add an interest, please scroll to the top of the page, click "add interest" and select "Patents")**

No, I have no relevant interests of this type

j. **Fiduciary Officer or Other Board Membership (If you need to add an interest, please scroll to the top of the page, click "add interest" and select "Fiduciary Officer")**

No, I have no relevant interests of this type

k. **Stock or stock options (If you need to add an interest, please scroll to the top of the page, click "add interest" and select the appropriate interest type)**

No, I have no relevant interests of this type

l. **Support for attending meetings or other travel (If you need to add an interest, please scroll to the top of the page, click "add interest" and select "Travel")**

No, I have no relevant interests of this type

4. **Was any individual paid to provide professional writing assistance with this manuscript?**

   No.

5. **Have you or your institution received equipment, materials, drugs, or services in direct support of the work in the manuscript (without time limit) not disclosed above?**

   No.

6. **In the past 36 months, have you received equipment, materials, drugs, medical writing, gifts or other services from for-profit or not-for-profit third parties whose interests may be affected by the content of the manuscript not disclosed above?**

   No.

7. **Are there other financial or non-financial interests that readers could perceive to have influenced, or that give the appearance of potentially influencing, what you wrote in the submitted work not disclosed above.**

   No.

Certification

I certify that I have answered every question and the information provided in this disclosure is complete and accurate.

Gauravi Mishra

Discloser Identifier: 126251195

Disclosure Purpose: VIHRI-CEEWAA-2024-0288

Employment Information: Currently Employed

Summary of Interests

I do not have any interests to disclose at this time.

Additional Questions

1. **Please select which of the following apply to each relationship or activity:**

   You are not disclosing any interests to this organization.

2. **I confirm I have disclosed all direct support for the present manuscript (e.g. funding, provision of study materials, medical writing, article processing charges, etc.) There is no time limit for this item.**

   Yes

3. **Please indicate below whether in the past 36 months you have had any of the following interests that are topically related to the work reported in the manuscript.**

   a. **Employment (If you need to add an interest, please scroll to the top of the page, click "add interest" and select “Employment”)**

      No, I have no relevant interests of this type

   b. **Grants or contracts for research (If you need to add an interest, please scroll to the top of the page, click "add interest" and select “Grant/Contract”)**

      No, I have no relevant interests of this type

   c. **Payment for consulting (If you need to add an interest, please scroll to the top of the page, click "add interest" and select “Independent Contractor”)**

      No, I have no relevant interests of this type

   d. **Payments or honoraria for lectures, presentations, speakers bureaus, or educational events (If you need to add an interest, please scroll to the top of the page, click "add interest" and select "Independent Contractor" and include the correct information under "Consultant")**

      No, I have no relevant interests of this type

   e. **Payment for service on an advisory board (If you need to add an interest, please scroll to the top of the page, click "add interest" and select “Independent Contractor,” and choose “Other”)**

      No, I have no relevant interests of this type

   f. **Payment for participation Data and safety monitoring board (If you need to add an interest, please scroll to the top of the page, click "add interest" and select “Independent Contractor”)**

      No, I have no relevant interests of this type

   g. **Expert witness testimony (If you need to add an interest, please scroll to the top of the page, click "add interest" and select “Independent Contractor”)**

      No, I have no relevant interests of this type

   h. **Royalties from Patents, Trademarks, Copyrights or other intellectual property (If you need to add an interest, please scroll to the top of the page, click "add interest" and select the appropriate interest type)**

No, I have no relevant interests of this type

i. **Patents planned, issued, or pending, whether or not you receive royalties (If you need to add an interest, please scroll to the top of the page, click "add interest" and select “Patents”)**

No, I have no relevant interests of this type

j. **Fiduciary Officer or Other Board Membership (If you need to add an interest, please scroll to the top of the page, click "add interest" and select “Fiduciary Officer”)**

No, I have no relevant interests of this type

k. **Stock or stock options (If you need to add an interest, please scroll to the top of the page, click "add interest" and select the appropriate interest type)**

No, I have no relevant interests of this type

l. **Support for attending meetings or other travel (If you need to add an interest, please scroll to the top of the page, click "add interest" and select “Travel”)**

No, I have no relevant interests of this type

4. **Was any individual paid to provide professional writing assistance with this manuscript?**

   No.

5. **Have you or your institution received equipment, materials, drugs, or services in direct support of the work in the manuscript (without time limit) not disclosed above?**

   No.

6. **In the past 36 months, have you received equipment, materials, drugs, medical writing, gifts or other services from for-profit or not-for-profit third parties whose interests may be affected by the content of the manuscript not disclosed above?**

   No.

7. **Are there other financial or non-financial interests that readers could perceive to have influenced, or that give the appearance of potentially influencing, what you wrote in the submitted work not disclosed above.**

   No.

Certification

I certify that I have answered every question and the information provided in this disclosure is complete and accurate.

Convey Global Disclosure System | AAMC

Richard Muwonge

Discloser Identifier: 126251212

Disclosure Purpose: VIHRI-CEEWAA-2024-0288

Employment Information: Currently Employed

Summary of Interests

Company or Organization

Entity	Type	Relevant to this Disclosure
World Health Organization	Employment Current Employment	
**Title:** Scientist		

Additional Questions

1. **Please select which of the following apply to each relationship or activity:**

   a. **Employment** World Health Organization

      The relationship is in direct support of the work reported in the manuscript anytime from when the work was conceived

2. **I confirm I have disclosed all direct support for the present manuscript (e.g. funding, provision of study materials, medical writing, article processing charges, etc.) There is no time limit for this item.**

   Yes

3. **Please indicate below whether in the past 36 months you have had any of the following interests that are topically related to the work reported in the manuscript.**

   a. **Employment (If you need to add an interest, please scroll to the top of the page, click "add interest" and select "Employment")**

      No, I have no relevant interests of this type

   b. **Grants or contracts for research (If you need to add an interest, please scroll to the top of the page, click "add interest" and select "Grant/Contract")**

      No, I have no relevant interests of this type

   c. **Payment for consulting (If you need to add an interest, please scroll to the top of the page, click "add interest" and select "Independent Contractor")**

      No, I have no relevant interests of this type

   d. **Payments or honoraria for lectures, presentations, speakers bureaus, or educational events (If you need to add an interest, please scroll to the top of the page, click "add interest" and select "Independent Contractor" and include the correct information under "Consultant")**

      No, I have no relevant interests of this type

   e. **Payment for service on an advisory board (If you need to add an interest, please scroll to the top of the page, click "add interest" and select "Independent Contractor," and choose "Other")**

      No, I have no relevant interests of this type

f. **Payment for participation Data and safety monitoring board (If you need to add an interest, please scroll to the top of the page, click "add interest" and select "Independent Contractor")**

No, I have no relevant interests of this type

g. **Expert witness testimony (If you need to add an interest, please scroll to the top of the page, click "add interest" and select "Independent Contractor")**

No, I have no relevant interests of this type

h. **Royalties from Patents, Trademarks, Copyrights or other intellectual property (If you need to add an interest, please scroll to the top of the page, click "add interest" and select the appropriate interest type)**

No, I have no relevant interests of this type

i. **Patents planned, issued, or pending, whether or not you receive royalties (If you need to add an interest, please scroll to the top of the page, click "add interest" and select "Patents")**

No, I have no relevant interests of this type

j. **Fiduciary Officer or Other Board Membership (If you need to add an interest, please scroll to the top of the page, click "add interest" and select "Fiduciary Officer")**

No, I have no relevant interests of this type

k. **Stock or stock options (If you need to add an interest, please scroll to the top of the page, click "add interest" and select the appropriate interest type)**

No, I have no relevant interests of this type

l. **Support for attending meetings or other travel (If you need to add an interest, please scroll to the top of the page, click "add interest" and select "Travel")**

No, I have no relevant interests of this type

4. **Was any individual paid to provide professional writing assistance with this manuscript?**

   No.

5. **Have you or your institution received equipment, materials, drugs, or services in direct support of the work in the manuscript (without time limit) not disclosed above?**

   No.

6. **In the past 36 months, have you received equipment, materials, drugs, medical writing, gifts or other services from for-profit or not-for-profit third parties whose interests may be affected by the content of the manuscript not disclosed above?**

   No.

7. **Are there other financial or non-financial interests that readers could perceive to have influenced, or that give the appearance of potentially influencing, what you wrote in the submitted work not disclosed above.**

   No.

Certification

I certify that I have answered every question and the information provided in this disclosure is complete and accurate.
