# Supplementary material for: Breast Cancer Screening Using Clinical Breast Examination: A Cost-Effectiveness Analysis for South Africa
Source: Value Health Reg Issues. 2025 Sep;49:None. doi: 10.1016/j.vhri.2025.101127 (PMC12411605; doi:10.1016/j.vhri.2025.101127)
Supplement: Supplemental Material [file mmc2.pdf]

Supplement to "Breast cancer screening using clinical breast examination: A cost-effectiveness analysis for South Africa”

Text S1: Part 1 – Decision tree

After an initial CBE at a PHC, women found to have abnormalities were referred to a higher-level facility for an additional CBE; based on the abnormalities identified at each step and available resources, these women possibly underwent an ultrasound followed by mammography and biopsy by fine-needle aspiration cytology or ultrasound-guided core or stereotactic core biopsy. After each screening round, women who were found to have no abnormalities or cancer were re-entered into the model for rescreening in the next round. Women with a missed BC diagnosis in any screening round re-entered the model for potential detection in subsequent screening rounds only if they were within the sojourn time, defined as the interval between the onset of preclinical screen-detectable disease and the onset of symptomatic (clinically detectable) disease.[1]

Text S2: Systematic literature review

We conducted a literature search for RCTs to determine the proportion of patients diagnosed with late-stage BC (stages III-IV) after CBE screening and how this proportion compared with that of patients with no screening, thus allowing us to estimate the effectiveness of CBEs for downstaging BC. We searched PubMed to identify studies published between 1 January 2005 and 13 July 2020 via a search strategy adapted from the Cochrane Breast Cancer Group Specialized Register.[2] The search strategy is reported in Supplementary Texts S3 and S4. We included RCTs conducted in LMICs that had at least two screening rounds because the first round of screening detected a greater number of prevalent cases than incident cases. If a

published study included fewer than two screening rounds but the RCT was ongoing, we contacted the authors to source data on additional rounds if available. RCTs were excluded if they met the following criteria: 1) included women with a previous history of BC; 2) included only male participants; 3) did not use CBEs as the sole screening modality; 4) reported mortality as the only outcome of interest; and 5) were published in languages other than English. The detailed inclusion and exclusion criteria are provided in Supplementary Table S1.

Text S3: Literature search

The search terms for CBE (*clinical breast exam, breast exam, and clinical exam*) and screening (*early detection, early diagnosis, breast cancer control, screening, and early detection of cancer*) were included. The BC, CBE, screening, and controlled trial search terms were combined using the Boolean operator “AND” to maximise the specificity of the search.

Text S4: PubMed search strategy

1. Breast Neoplasms[MeSH Terms]

2. (Neoplasms, Ductal, Lobular, and Medullary[MeSH Terms])

3. Fibrocystic Breast Disease[MeSH Terms]

4.1 OR 2 OR 3

5. Breast[MeSH Terms]

6. Breast[Text Word]

7. 5 OR 6

8. breast milk[Text Word]

9. (breast[Text Word]) AND tender*[Text Word]

10. breast feeding[Text Word]

11. 8 OR 9 OR 10

12. 7 NOT 11

13. Neoplasms[MeSH Terms]

14. 12 AND 13

15. Lymphedema[MeSH Terms]

16. 12 AND 15

17. (((breast[Text Word]) OR mammary[Text Word]) AND cancer*[Text Word])

18. 4 OR 14 OR 16 OR 17

19. Breast[Text Word]) AND screen*[Text Word]

20. Clinical breast exam*[Text Word]

21. Breast exam*[Text Word]

22. Clinical exam*[Text Word]

23. 19 OR 20 OR 21 OR 22

24.18 AND 23

25. Early detection[Text Word]

26. Early diagnosis[Text Word]

27. Breast cancer control[Text Word]

28. Mass Screening[MeSH Terms]

29. Early Detection of Cancer[MeSH Terms]

30. Screening[Text Word]

31. 25 OR 26 OR 27 OR 28 OR 29 OR 30

32. 24 AND 31

33. "clinical trial"[Publication Type]

34. Clinical Trials[MeSH Terms]

35 "comparative study"[Publication Type]

36. "randomized controlled trial"[Publication Type]

37. "controlled clinical trial"[Publication Type]

38. randomized[TIAB]

39. placebo[TIAB]

40. "clinical trials as topic"[MeSH Terms]

41. randomly[TIAB]

42. trial[Title]

43. (crossover[Text Word]) OR cross-over[Text Word]

44. "pragmatic clinical trials as topic"[MeSH Terms]

45. "pragmatic clinical trial"[Publication Type]

46. preliminary results[Text Word]

47. 33 OR 34 OR 35 OR 36 OR 37 OR 38 OR 39 OR 40 OR 41 OR 42 OR 43 OR 44 OR 45 OR 46

48. (animals[MeSH Terms]) NOT humans[MeSH Terms]

49. 47 NOT 48

50. 32 AND 49

Table S1: Inclusion and exclusion criteria

**Selection Criteria**	**Inclusion**	**Exclusion**
Population	• Women of any age who had not been diagnosed with breast cancer<br>• Both male and female participants	• Women with a previous history of breast cancer.<br>• Only male participants
Intervention	• Clinical breast examination alone	• Mammography<br>• Education on breast cancer<br>• Breast self-examination<br>• Modalities other than clinical breast examinations
Comparators	• No intervention<br>• Usual care<br>• Education on breast cancer	NA

Selection Criteria	Inclusion	Exclusion
Outcomes	• Proportion of patients diagnosed with late-stage breast cancer (stages III-IV)<br>• Proportion of patients diagnosed with late-stage breast cancer (as defined by the authors)	• Mortality as the only reported outcome of interest<br>• Other outcomes
Study type	• Randomised clinical trials including cluster randomised trials<br>• Case–controlled trials	• Systematic reviews
Language	• Published in English	• Published in languages other than English
Other	• Available online<br>• Free to access<br>• LMICs	• Not available online<br>• Countries that are not LMICs

We identified two cluster RCTs that met our inclusion criteria (see Supplementary Figure S1 for the results of the screening process). One of the studies, that by Sankaranarayanan et al., carried out in Kerala, India, was initiated in January 2006, with follow-up ending in December 2019.[3,4] At the time of this literature search, the results from only one round of screening had been published, but the authors of that study provided unpublished data for rounds 2 and 3 of screening from the file system at the IARC. The second study was conducted in Mumbai, India, where participants were recruited beginning in May 1998. When the trial ended in March 2019, four screening rounds at 2-year intervals, followed by 13 years of follow-up, had been completed.[5,6] The study characteristics and screening protocols of each study are summarised in Supplementary Table S2. The number of women in each study arm by age group can be found in Supplementary Table S3.

Figure S1: Flow diagram of the study inclusion process

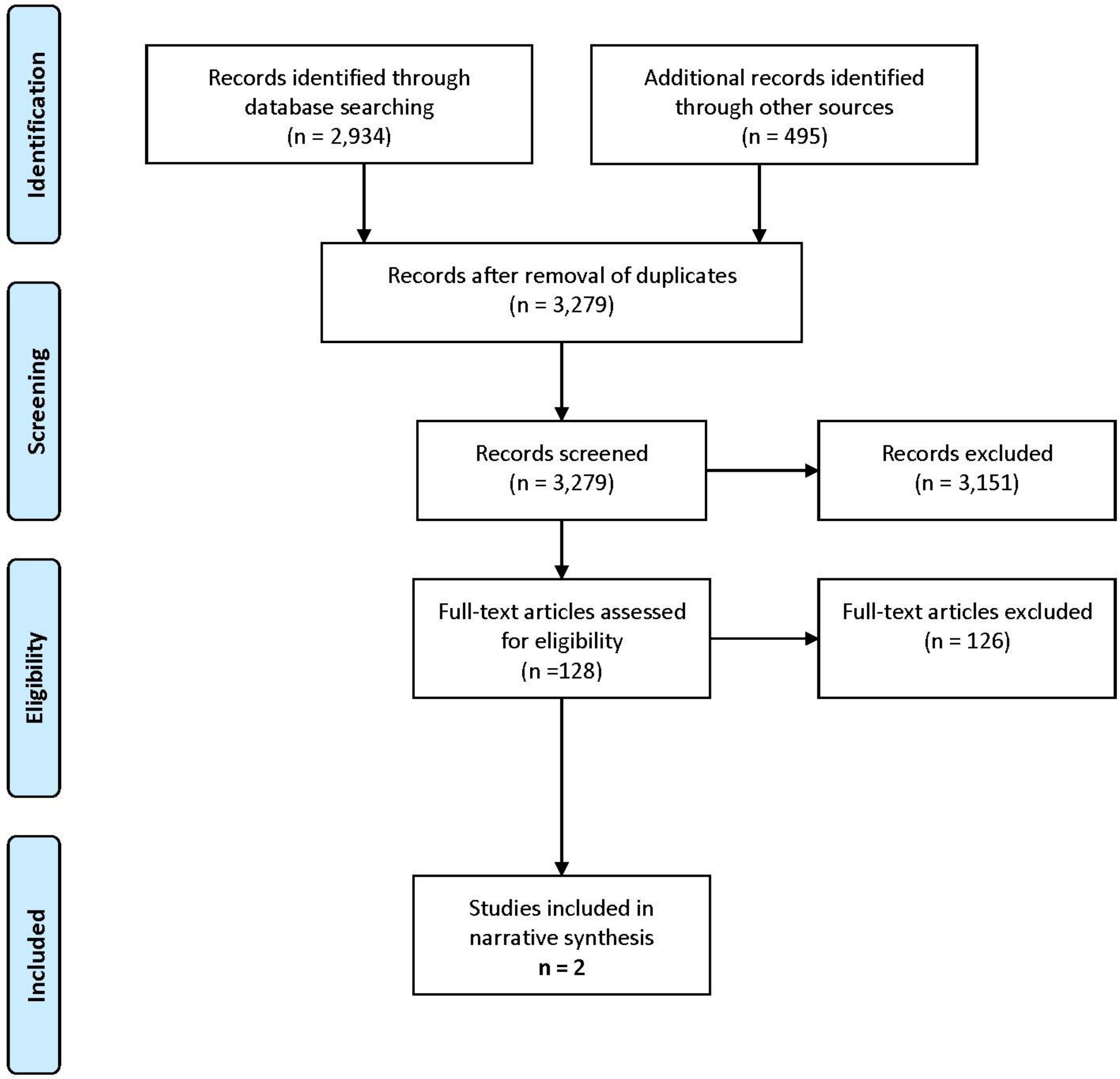

Table S2: Trial data - Summary of included study characteristics and screening protocols

First author (year)	Duration of trial	Setting or population (intervention, n; control, n)	Enrolment age, years	Study design	Intervention	Control	Primary outcomes	Intermediate outcomes	Inter-screening interval (months)	Screening rounds completed	Total screening rounds
Sankaranarayanan (2011)<br><br>IARC+ data on file	2006 - 2019	Trivandrum district (Kerala, India); 275 electoral wards (clusters) (55,843; 59,447)	30 - 69	RCT; cluster randomisation of 275 clusters - 133 clusters in the intervention arm & 141 clusters in the control arm.	CBE carried out by a trained female healthcare worker.	Health education	Effect on mortality and incidence	Stage distribution	36	3	3
Mittra (2021)	1998 - 2018	Mumbai (India); 20 slum clusters (75,360; 76,178)	35 - 64	RCT; cluster randomisation - 10 clusters in each arm.	CBE carried out by trained female primary healthcare providers and cancer awareness every 2 years.	One round of cancer awareness	Effect on mortality and incidence	Staging at diagnosis	24	4	4

+ International Agency for Research on Cancer

Table S3: Number of women in each study arm according to age band

	Study Arm		Control Arm	
**Age band**	**Sankaranarayanan (2011)**	**Mittra (2021)**	**Sankaranarayanan (2011)**	**Mittra (2021)**
40-44	8,120	17,311	8,690	17,245
45-49	8,153	14,183	8,729	14,111
50-54	6,887	9,422	7,385	9,564
55-59	6,013	6,049	6,540	6,127
60-64	5,304	5,494	5,706	6,218
65-69	5,098	n/a	5,366	n/a

The data collected from the two included RCTs included the number of patients in each study arm and the distribution of early-stage disease versus late-stage disease within each arm. Age-specific screen positivity rates and screening compliance rates (i.e., the proportion of the population that was intended to be screened and participated in screening) were sourced from the trial by Sankaranarayanan et al. using data stored at the International Agency for Research on Cancer (IARC).[3]

We used an intention-to-treat approach to determine the impact of CBE screening on downstaging. The distributions of BC by stage and age band for each of the study arms and for the age bands of <50 and 50-69 years are shown in Table S2. Assuming that any downstaging from late-stage (stages III and IV) to early-stage (stages I and II) BC was a result of CBE screening, we estimated the numbers and percentages of BCs that were downstaged. For example, based on the number of late-stage BC cases in women aged 50-69 years in the control arm, the number of BC cases we would expect in the study arm was as follows:

$$\frac{\text{Number of women randomised to the study arm} \times \text{Number of women with late} - \text{stage BC in the control arm}}{\text{Number of women randomised to the control arm}}$$

$$\text{Expected late} - \text{stage BC} = \frac{44{,}267 \times 142}{46{,}906} = 134$$

However, 121 late-stage BCs were observed in the two trials, and 13 (13/134) late-stage BCs were downstaged.

Table S4: Estimated percentage of women with downstaged BC on the basis of data from the Trivandrum and Mumbai trials[3,4,5]

	Control arm, no. (%)	Study arm, no. (%)	No. downstaged (actual minus expected late-stage BC)	Percentage downstaged
Women aged <50 years				
No. of women randomised to each arm	48,775	47,767		
Women with stage I-II BC	261 (55%)	319 (63%)		
Women with stage III-IV BC	212 (45%)	187 (37%)	21	10.10%
Women aged 50-69 years				
No. of women randomised to each arm	46,906	44,267		
Women with stage I-II BC	194 (58%)	224 (65%)		
Women with stage III-IV BC	142 (42%)	121 (35%)	13	9.7%

Table S5: Annual number of women screened, screen-detected cancers and 5-year detection rates according to the scale-up scenario

	2023	2024	2025	2026	2027	Screen-detected breast cancers	*Detection rate per 1,000 women screened*
Baseline (40-69, 5% intended to be screened annually)	223,570	226,475	229,418	231,183	234,188	2,513	*2.20*
Scenario 1 (40-69, slower scale-up)	223,570	452,950	688,255	924,733	1,170,938	8,278	*2.39*
Scenario 2 (40-69, aggressive scale-up)	223,570	1,019,137	1,835,346	2,658,607	3,512,815	19,280	*2.08*

Table S6: Annual proportion of eligible women targeted for screening, actual screening proportion adjusted for compliance (varying by age band), and percentage mortality reduction compared with baseline

	2023 % intended to be screened (% screened)	2024 % intended to be screened (% screened)	2025 % intended to be screened (% screened)	2026 % intended to be screened (% screened)	2027 % intended to be screened (% screened)	Percentage change in YLLs relative to baseline *(deaths)*
Baseline (40-69, 5% intended to be screened annually)	5% (3.5-3.6%)	5% (3.5-3.6%)	5% (3.5-3.6%)	5% (3.5-3.6%)	5% (3.5-3.6%)	-
Scenario 1 (40-69, slower scale-up)	5% (3.5-3.6%)	10% (6.9-7.2%)	15% (10.4-10.8%)	20% (13.8-14.4%)	25% (17.3-18%)	0.71% (0.72%)
Scenario 2 (40-69, aggressive scale-up)	5% (3.5-3.6%)	23% (15.6-16.2%)	40% (27.7-28.8%)	58% (39.7-41.3%)	75% (51.8-53.9%)	2.25% (2.26%)

YLLs - years of life lost

Table S7: Discounted costs per woman screened (USD 2022)

Age band	**Baseline** (40-69, 5% targeted annually)	**Scenario 1** (40-69, slower scale-up)	**Scenario 2** (40-69, aggressive scale-up)
**40-44**	37.33	37.47	37.43
**45-49**	36.33	36.48	36.42
**50-54**	28.87	29.02	28.92
**55-59**	26.59	26.74	26.62
**60-64**	22.99	23.13	22.98
**65-69**	17.52	17.66	17.50
**All ages**	**30.11**	**30.27**	**30.17**

Figure S2: Cost-effectiveness acceptability curve

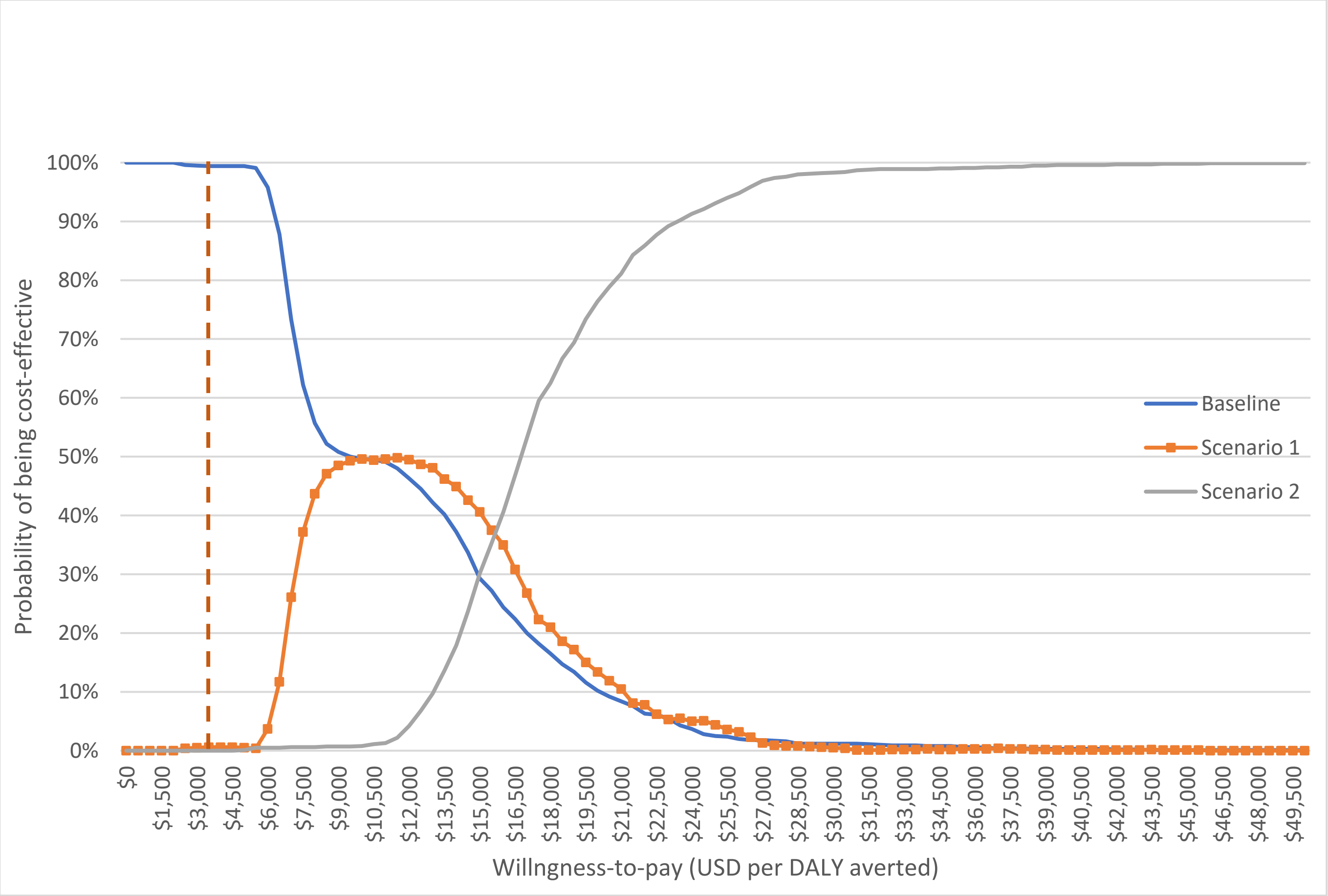

References

1. Cheung S, Hutton JL, Brettschneider JA. Review of sojourn time calculation models used in breast cancer screening. 2017;(17):1-21.

2. Cochrane Breast Cancer. Specialised Register. Accessed July 15, 2020, https://breastcancer.cochrane.org/specialised-register

3. Ramadas K, Basu P, Matthew BS, et al. Effectiveness of triennial screening with clinical breast examination: 14 - years follow - up outcomes of randomized clinical trial in Trivandrum, India. *Cancer*. 2023;(June 2022):272-282. doi:10.1002/cncr.34526

4. Sankaranarayanan R, Ramadas K, Thara S, et al. Clinical breast examination: Preliminary results from a cluster randomized controlled trial in India. *J Natl Cancer Inst*. 2011;103(19):1476-1480. doi:10.1093/jnci/djr304

5. Mittra I, Mishra GA, Dikshit RP, et al. Effect of screening by clinical breast examination on breast cancer incidence and mortality after 20 years: Prospective, cluster randomised controlled trial in Mumbai. *The BMJ*. 2021;372:1-9. doi:10.1136/bmj.n256

6. Mittra I, Mishra GA, Singh S, et al. A cluster randomized, controlled trial of breast and cervix cancer screening in Mumbai, India: methodology and interim results after three rounds of screening. *International journal of cancer*. 2010/2// 2010;126(4):976-984. doi:10.1002/ijc.24840
